# Evaluating ChatGPT-4’s Accuracy in Identifying Final Diagnoses Within Differential Diagnoses Compared With Those of Physicians: Experimental Study for Diagnostic Cases

**DOI:** 10.2196/59267

**Published:** 2024-06-26

**Authors:** Takanobu Hirosawa, Yukinori Harada, Kazuya Mizuta, Tetsu Sakamoto, Kazuki Tokumasu, Taro Shimizu

**Affiliations:** 1 Department of Diagnostic and Generalist Medicine Dokkyo Medical University Tochigi Japan; 2 Department of General Medicine Okayama University Graduate School of Medicine, Dentistry and Pharmaceutical Sciences Okayama Japan

**Keywords:** decision support system, diagnostic errors, diagnostic excellence, diagnosis, large language model, LLM, natural language processing, GPT-4, ChatGPT, diagnoses, physicians, artificial intelligence, AI, chatbots, medical diagnosis, assessment, decision-making support, application, applications, app, apps

## Abstract

**Background:**

The potential of artificial intelligence (AI) chatbots, particularly ChatGPT with GPT-4 (OpenAI), in assisting with medical diagnosis is an emerging research area. However, it is not yet clear how well AI chatbots can evaluate whether the final diagnosis is included in differential diagnosis lists.

**Objective:**

This study aims to assess the capability of GPT-4 in identifying the final diagnosis from differential-diagnosis lists and to compare its performance with that of physicians for case report series.

**Methods:**

We used a database of differential-diagnosis lists from case reports in the *American Journal of Case Reports*, corresponding to final diagnoses. These lists were generated by 3 AI systems: GPT-4, Google Bard (currently Google Gemini), and Large Language Models by Meta AI 2 (LLaMA2). The primary outcome was focused on whether GPT-4’s evaluations identified the final diagnosis within these lists. None of these AIs received additional medical training or reinforcement. For comparison, 2 independent physicians also evaluated the lists, with any inconsistencies resolved by another physician.

**Results:**

The 3 AIs generated a total of 1176 differential diagnosis lists from 392 case descriptions. GPT-4’s evaluations concurred with those of the physicians in 966 out of 1176 lists (82.1%). The Cohen κ coefficient was 0.63 (95% CI 0.56-0.69), indicating a fair to good agreement between GPT-4 and the physicians’ evaluations.

**Conclusions:**

GPT-4 demonstrated a fair to good agreement in identifying the final diagnosis from differential-diagnosis lists, comparable to physicians for case report series. Its ability to compare differential diagnosis lists with final diagnoses suggests its potential to aid clinical decision-making support through diagnostic feedback. While GPT-4 showed a fair to good agreement for evaluation, its application in real-world scenarios and further validation in diverse clinical environments are essential to fully understand its utility in the diagnostic process.

## Introduction

### Diagnostic Error and Feedback

A well-developed diagnostic process is fundamental to medicine. Diagnostic errors [1], which include missed, incorrect, or delayed diagnoses [2], result in severe misdiagnosis-related harm, affecting up to 795,000 patients annually in the United States [3]. These errors often stem from a failure to correctly identify an underlying condition [4,5]. Enhancing the diagnostic process is crucial, with diagnostic feedback playing a key role [6]. The feedback enables physicians to assess their diagnostic accuracy and adjust their subsequent clinical decisions accordingly [7]. Common diagnostic feedback methods include self-reflection [8,9], peer review [1], and clinical decision support systems (CDSSs), which aim to enhance decision-making at the point of care [10]. Unlike the retrospective nature of self and peer review processes, feedback from CDSSs is provided in real-time [11], offering immediate support and guidance during the diagnostic process. This timely feedback is particularly advantageous in fast-paced clinical settings where timely decision-making is critical.

### CDSSs and Artificial Intelligence

CDSSs are categorized into 2 main types: knowledge-based and nonknowledge-based systems [10]. Knowledge-based CDSSs rely on established medical knowledge including clinical guidelines, expert protocols, and information on drug interactions. In contrast, nonknowledge-based systems, particularly those using artificial intelligence (AI), leverage advanced algorithms, machine learning, and statistical pattern recognition. Unlike their rule-based counterparts, these systems adapt over time, continuously refining their insights and recommendations. The rapid integration of AI into CDSSs highlights the growing importance of advanced technologies in health care [12]. In recent years, generative AI through large language models (LLMs) has been reshaping health care, offering improvements in diagnostic accuracy, treatment planning, and patient care [13,14]. AI systems, emulating human cognition, continuously learn from new data [15]. They assist health care professionals by analyzing complex patient data, thereby enhancing clinical decision-making and patient outcomes [10].

### Growing Importance of Generative AI

In this context of rapidly integrating AI into CDSSs, generative AIs have marked a new era in digital health. LLMs are advanced AI algorithms trained on extensive textual data, enabling them to process and generate human-like text, thereby providing valuable insights to medical diagnostics. Several generative AI tools are now available to the public, including Bard (currently Gemini) by Google [16,17], LLM Meta AI 2 (LLaMA2) by Meta AI [18], and ChatGPT, developed by OpenAI [19]. These AI tools, which use LLMs, have successfully passed national medical licensing exams without specific training or reinforcement [20], demonstrating their potential in medical diagnostics. Among these, ChatGPT stands out as one of the most extensively researched generative AI applications in health care [21]. Specifically, in diagnostics, a recent study has shown that these generative AI systems, particularly ChatGPT with GPT-4, demonstrate excellent diagnostic capability when answering clinical vignette questions [22]. Additionally, other studies, including our own, have assessed AI systems’ performance in one aspect of the diagnostic process, generating differential diagnosis lists [23-25]. While broader studies compare a variety of state-of-the-art models, our analysis focuses on the distinct capabilities and impacts of these specific tools within medical diagnostics.

### Generative AI Systems in the Diagnostic Process

The diagnostic process involves collecting clinical information, forming a differential diagnosis, and refining it through continuous feedback [26]. This feedback consists of patient outcomes, test results, and final diagnoses [27,28]. Similar to traditional CDSSs, generative AI systems can enhance this feedback loop [29]. However, a gap previously existed in the systematic comparison of differential diagnoses with final diagnoses through a feedback loop [27]. Given this background, it remains less explored how effectively these AI systems integrate their feedback into clinical workflow. To address this gap, exploring how generative AI systems provide feedback by comparing final diagnoses with differential-diagnosis lists represents a straightforward and viable first step. This study used differential diagnosis lists to assess diagnostic accuracy. This approach was chosen to mimic a key aspect of the clinical decision-making process, where physicians often narrow down a broad list of potential diagnoses to determine the most likely one. This method reflects a critical use case for AI in health care, potentially speeding up and refining diagnostic accuracy. In our previous short communication, we reported that the fourth generation ChatGPT (GPT-4) showed very good agreement with physicians in evaluating the lists for a limited number of case reports published from our General Internal Medicine (GIM) department [30]. Building on this research, this study focused on assessing the capability of GPT-4 in identifying the final diagnosis from differential-diagnosis lists for comprehensive case report series, compared with those of physicians. Furthermore, this research aimed to demonstrate the role of generative AI, particularly GPT-4, in enhancing the diagnostic learning cycle through effective feedback mechanisms.

## Methods

### Overview

**Figure 1:**
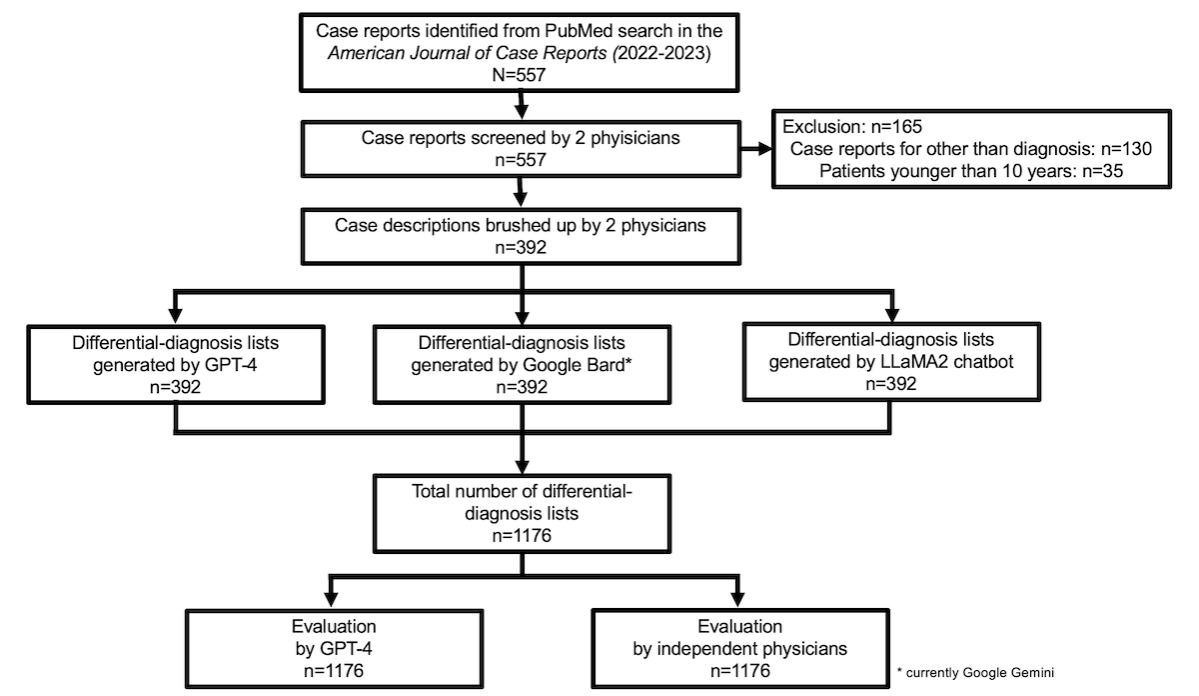
Study flowchart of inclusion of case reports, generation of differential-diagnosis lists, and evaluation of the lists. LLaMA2: LLM Meta AI 2.

We conducted an experimental study using GPT-4 and the differential-diagnosis lists generated by 3 AI systems inputting into case descriptions. The research was conducted at the Department of Generalist and Diagnostic Medicine (GIM), Dokkyo Medical University, Tochigi, Japan. Our research methodology encompassed preparing a data set for differential-diagnosis lists and the corresponding final diagnoses, assessing these lists using GPT-4, and having physicians evaluate the lists. Figure 1 illustrates this study flow.

### Ethical Considerations

Since we used a database extracted from published case reports, obtaining ethical approval was not applicable.

### Database of Differential-Diagnosis Lists and Final Diagnoses

We used our data set from a previous study (TH, YH, KM, T Sakamoto, KT, T Shimizu. Diagnostic performance of generative artificial intelligences for a series of complex case reports. unpublished data, November 2023). From the PubMed search, we identified a total of 557 case reports. We excluded the nondiagnosed cases (130 cases) and the pediatric cases, aged younger than 10 years (35 cases). The exclusion criteria were based on the previous research for CDSS [31]. After the exclusion, we included 392 case reports. The case reports were brushed up as case descriptions to focus on the diagnosis. The authors typically defined the final diagnoses. Through inputting into the case descriptions and systematic prompt, 3 generative AI systems—GPT-4, Google Bard (currently Google Gemini), and LLaMA2 chatbot—generated the top 10 differential-diagnosis lists. The AI systems used were not trained for any additional medical use or reinforced. The main investigator (TH) conducted the entire process, with validation provided by another investigator (YH). Through this process, this data set included differential diagnosis lists corresponding to case descriptions and final diagnoses from case reports in the *American Journal of Case Reports*. Detailed lists of differential diagnoses and their final diagnoses are shown in Multimedia Appendix 1.

### GPT-4 Assessment of the Differential-Diagnosis Lists

In selecting the generative AI systems for evaluation, we focused on GPT-4 due to its distinct architectural frameworks and widespread use in the field of health care research. GPT-4, developed by OpenAI, is notable for its advanced natural language processing capabilities and extensive training data set, making it particularly relevant for health care [32]. We used the August 3 version and September 25 version of GPT-4 to evaluate differential diagnosis lists. The access date was from September 11, 2023, to October 6, 2023. A structured prompt was crafted to ascertain whether GPT-4 could identify the final diagnosis within a list and its position if present. The prompt required direct copying and pasting of the final diagnoses and differential diagnosis lists from our data set. We assessed the inclusion of the final diagnosis in the list (Yes=1, No=0) and its position. The prompt selection was a preliminary investigation. To ensure unbiased output, each session was isolated by deactivating chat history and training controls and restarting GPT-4 before every new evaluation. We obtained a single output from GPT-4 for each differential diagnosis list. The details of this structured prompt in this study are expounded in Multimedia Appendix 2.

### Physician Assessment of the Differential-Diagnosis Lists

For comparison, 2 independent physicians (KM and T Sakamoto) also evaluated the differential diagnosis lists. The presence of the final diagnosis within the differential diagnosis lists was marked with a 1 or 0. A “1” was marked when the lists precisely and acceptably identified the final diagnosis [33], further ranking it from 1 to 10 based on its placement. A “0” indicated its absence. Discrepancies between the evaluations of the 2 physicians were resolved by another physician (KT). Notably, the physicians were blinded to which AI generated the lists they assessed. We selected 3 independent physicians, specializing in GIM. Selection was based on expertise in diagnostic processes and familiarity with AI technologies in health care. All physicians underwent a brief guidance session to familiarize themselves with the evaluation criteria and objectives of the study to ensure consistent assessment standards.

### Outcome

The primary outcome was defined as the κ coefficient for interrater agreement between GPT-4 and the physicians’ evaluations for the differential-diagnosis lists generated by 3 AI systems including GPT-4, Google Bard (currently Google Gemini), and LLaMA2 chatbot. The secondary outcomes were defined as the κ coefficients for interrater agreement between GPT-4 and the physicians’ evaluations for the differential diagnosis lists generated by each AI system. Additionally, another secondary outcome was defined as the ranking patterns between GPT-4’s evaluation and that of physicians.

### Statistical Analysis

Analytical procedures were conducted using R (version 4.2.2; The R Foundation for Statistical Computing). The agreement between different evaluations was quantified using the Cohen κ coefficient through the irr package in R. Agreement strength was categorized as per Cohen κ benchmarks: values under 0.40 indicated poor agreement; values between 0.41 and 0.75 showed fair to good agreement; and values ranging from 0.75 to 1.00 denoted very good agreement [34]. The 95% CIs were used to quantify uncertainty. Additionally, we compared ranking patterns between GPT-4’s evaluation and that of physicians [35].

## Results

### Overall Evaluation

**Figure 2:**
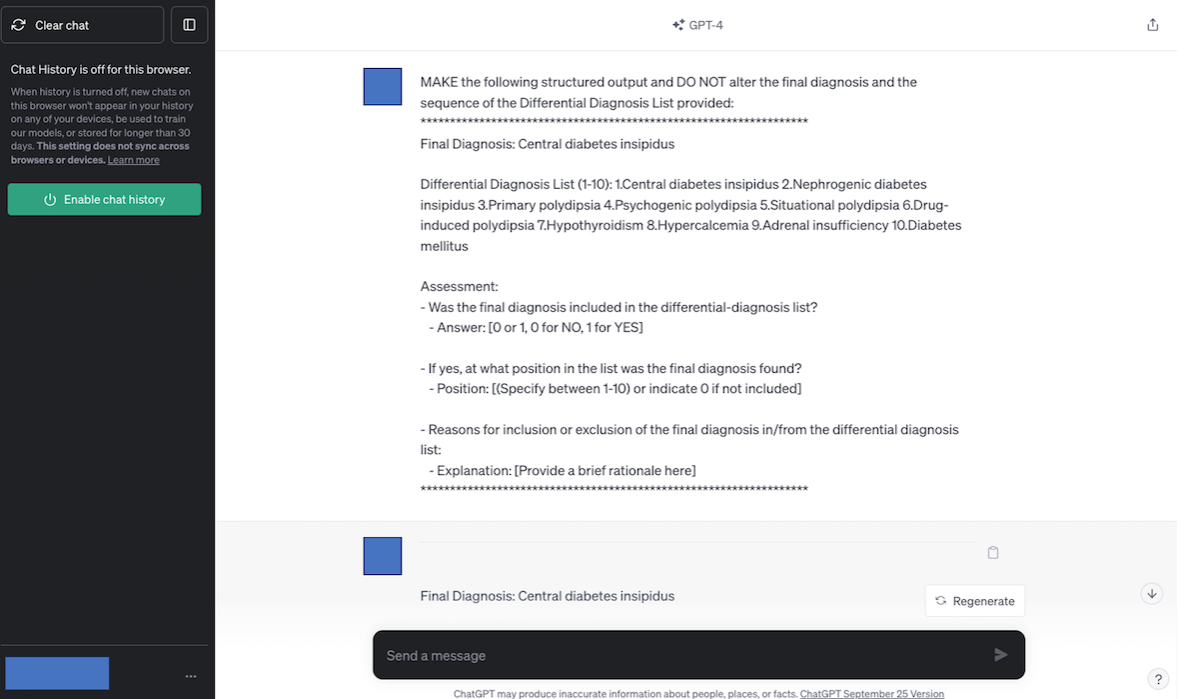
The representative input for GPT-4 generated to evaluate whether the final diagnosis was included in the differential diagnosis.

**Figure 3:**
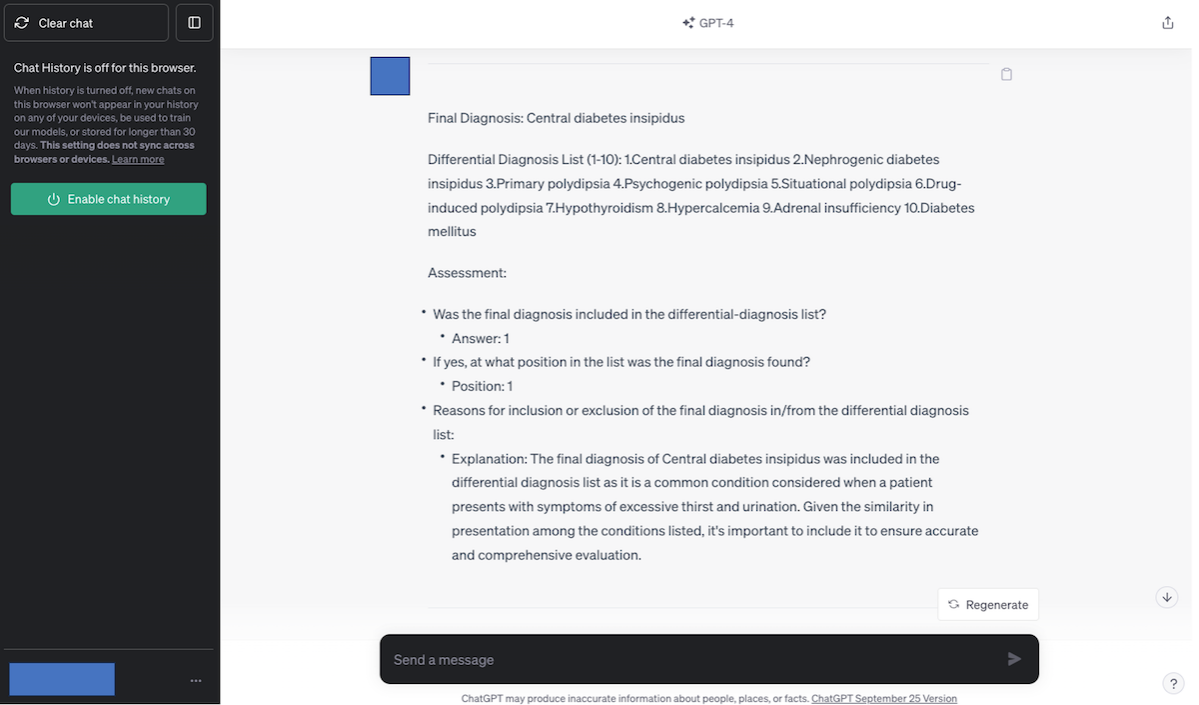
From the input (Figure 2), GPT-4 generated the representative output of its evaluation.

This study involved 3 generative AI systems—GPT-4, Google Bard (currently Google Gemini), and LLaMA2 chatbot—outputting differential-diagnosis lists for 392 case descriptions, resulting in a total of 1176 lists. In 825 lists where physicians included a final diagnosis, GPT-4 matched 636 lists and did not match 189 lists. Conversely, in 351 lists where physicians did not include a final diagnosis, GPT-4 matched 330 lists and did not match 21 lists. In total, GPT-4’s evaluations matched the physicians’ evaluations in 966 out of 1176 lists (82.1%). Cohen κ coefficient was 0.63 (95% CI 0.56-0.69), indicating a fair to good agreement between GPT-4 and the physicians’ evaluations. GPT-4 omitted the final diagnosis in 16.1% (n=189) of cases, contrasting with physicians’ evaluations that included these diagnoses. Table 1 shows GPT-4’s evaluations concurred with the physicians’ evaluations. Table 2 details the κ coefficient for interrater agreement between GPT-4 and the physicians’ evaluations. The representative input used in GPT-4’s evaluations is illustrated in Figure 2, and the corresponding output is shown in Figure 3. A formed data set is shown in Multimedia Appendix 3.

**Table 1:** GPT-4’s evaluations concurred with the physicians’ evaluations.

Variables	GPT-4		Total (N=1176)
	Matched	Did not match	
Inclusion of final diagnosis	636	189	825
Noninclusion of final diagnosis	330	21	351

**Table 2:** κ coefficient for interrater agreement between GPT-4 and the physicians’ evaluations for the differential diagnosis lists.

Differential-diagnosis lists generator	Cohen κ coefficient (95% CI)	Strength of agreement [34]	Number of differential-diagnosis lists
All	0.63 (0.56-0.69)	Fair to good	1176
GPT-4	0.47 (0.39-0.56)	Fair to good	392
Google Bard^a^	0.67 (0.52-0.73)	Fair to good	392
LLaMA2 chatbot^b^	0.63 (0.52-0.73)	Fair to good	392

^a^Currently Google Gemini.

^b^LLaMA2: LLM Meta AI 2.

### Evaluation of Each Generative AI

The κ coefficients for differential-diagnosis lists generated by GPT-4, Google Bard (currently Google Gemini), and LLaMA2 chatbot were 0.47 (95% CI 0.39-0.56), 0.67 (95% CI 0.52-0.73), and 0.63 (95% CI 0.52-0.73), respectively. All κ coefficients indicated a fair to good agreement between GPT-4 and the physicians’ evaluations.

### Comparison of Ranking Patterns Between GPT-4 and Physicians

**Figure 4:**
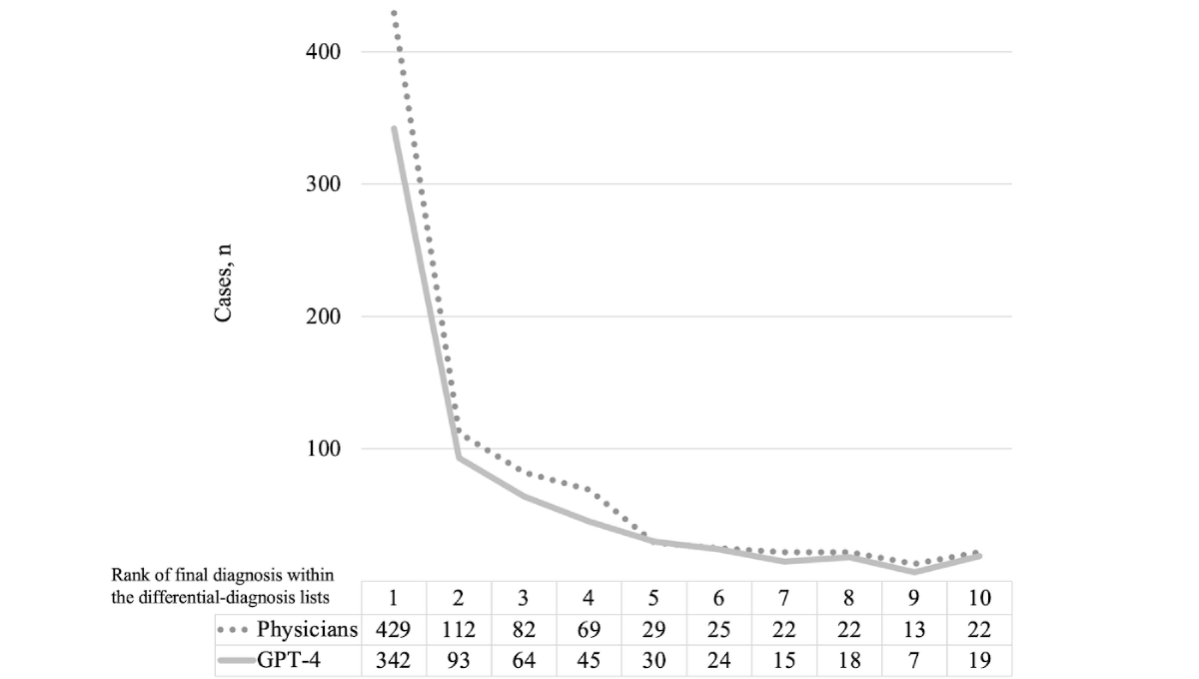
Comparison of ranking patterns in evaluations by GPT-4 and physicians.

Both GPT-4’s evaluation and that of physicians showed a general trend of decreasing frequency as the rank increases. Figure 4 shows the comparisons of ranking patterns between GPT-4 and physicians.

### Evaluation Between Physicians

Physicians’ evaluations (KM and T Sakamoto) for the differential diagnosis lists showed very good agreement, with concordance in 88.8% (n=1044) of cases. The κ coefficient was 0.75 (95% CI 0.46-0.99).

## Discussion

### Principal Results

This experimental study highlights several key findings. First, GPT-4’s evaluations matched those of physicians in more than 82% (n/N=966/1176) of the cases, demonstrating fair to good agreement according to κ coefficient values. These results imply that GPT-4’s accuracy in identifying the final diagnosis within differential-diagnosis lists is comparable to that of physicians. Unlike traditional CDSSs, generative AI systems, including GPT-4, are capable of performing multiple roles in the diagnostic process including formulating and assessing differential diagnoses. These capabilities highlight GPT-4’s potential to streamline diagnostics in clinical settings by expediting diagnostic feedback [36]. Our study design focuses on GPT-4’s ability to refine and validate pre-existing diagnostic considerations as supplementary tools for medical diagnostics. This scenario is akin to real-world clinical settings where generative AI systems could verify and support physicians’ final diagnostic decisions. By assessing the AI’s accuracy in this context, we can better understand its potential role and limitations in practical medical applications. Furthermore, in medical education, generative AI tools, like GPT-4, can offer students valuable self-learning opportunities. They provide timely feedback in the form of final diagnoses [37], enabling them to cross-reference with reliable sources for verification [38].

Second, GPT-4 failed to identify the final diagnosis in 16% (n/N=189/1176) of differential-diagnosis lists, even though these diagnoses were recognized by the evaluating physicians. Notably, despite achieving very good agreement among physicians, GPT-4 did not reach similar levels of concordance. This discrepancy highlights potential areas for improving the system’s ability to interpret and analyze complex medical data. This discrepancy arises primarily from GPT-4’s reliance on textual patterns and word associations within the provided differential diagnosis lists. Unlike physicians, who use a comprehensive medical knowledge base and clinical experience, an inherent limitation in generative AI systems like GPT-4 is their reliance on existing data patterns and textual association. To mitigate these discrepancies, continuous development in generative AI systems for health care is needed. Additionally, future research should focus on enhancing the medical training of these systems. This will enhance the generative AI systems’ diagnostic feedback, making it more adaptable to real clinical settings.

Third, regarding evaluation at what rank in the differential-diagnosis list was the final diagnosis found, both GPT-4 and physicians exhibited a trend of decreasing frequency. This suggests GPT-4’s diagnosis ranking shows a similar trend to physicians’ diagnosis ranking. Moreover, all 3 generative AI systems, including GPT-4, Google Bard (currently Google Gemini), and LLaMA2 chatbot, prioritized the most likely diagnoses at the top of the list, leading to a natural decrease in frequency as less-probable diagnoses are ranked lower. Therefore, generative AI systems showed the potential not only to generate differential diagnosis lists for clinical cases but also to evaluate these lists as feedback.

Fourth, an examination of the differential diagnosis lists generated by 3 different AI systems showed the overlap in the 95% CI for the κ coefficients across the 3 AI platforms. One might hypothesize that GPT-4 would exhibit improved performance when evaluating differential-diagnosis lists it generated itself. However, observed results may stem from the inherent variability in generative AI outputs including GPT-4. This inherent variability underscores the challenge of maintaining a consistent standard of accuracy and reliability in the outputs from generative AI systems. Even when evaluating differential-diagnosis lists generated by itself, GPT-4’s performance did not markedly surpass that of lists generated by other AI systems. Additionally, the observed performance differences may be partially due to version inconsistencies. The generation of differential diagnosis lists used an earlier version of GPT-4 (March 24). Subsequent evaluations used later versions (August 3 and September 25). Different versions of generative AI systems can exhibit varied capabilities and outputs, potentially impacting the accuracy and consistency of diagnostic evaluations. This highlights the need for ongoing updates and version alignment in clinical AI applications to maintain reliability.

### Limitations

This study has several limitations. First, GPT-4’s role was limited to identifying the final diagnosis within the differential diagnosis list. The current binary evaluation method has not been a well-established approach to evaluating diagnostic performance by other CDSSs. Another study used a 5-grade level of accuracy for a variety number of differentials [39]. Investigating more complex outcomes, such as quantitative evaluations and additional clinical suggestions, might yield different results. Second, our inputs to GPT-4 consisted only of the final diagnoses and the differential diagnosis list, without the case descriptions that generated these lists. Further research should examine what types of input enhance AI systems’ performance the most. Third, there was a nonnegligible risk associated with generative AI systems, including GPT-4, regarding their capacity to inadvertently learn from and replicate the information contained in publicly available case reports. Fourth, the data set was sourced from a single case reports journal and generated by 3 AI systems. Future research would benefit from using real-world scenarios [40]. Expanding the data set to include a more diverse range of AI systems is also advisable.

Regarding limitations for generative AI systems, like GPT-4, there is currently no approval for their use as CDSSs. Furthermore, GPT-4 operates as a fee-based application, which could potentially limit its accessibility to the wider public. Additionally, the reliability of generative AI systems can vary based on the input data it was trained on. If it is not exposed to diverse clinical scenarios during its training, it may not be as effective in real-world diagnostic situations [41]. Moreover, while AI tools can assist, they do not replace the nuanced judgments and decision-making processes of human physicians [42,43]. Additionally, the rapid evolution of AI means that our findings may become outdated as Google Bard and LLaMA2 were updated to the new LLM model, Google Gemini and LLaMA3, respectively [17,44]. Finally, overreliance on AI without critical review could lead to diagnostic errors [45].

### Comparison With Prior Work

In our previous study involving GPT-4 [30], we observed a very good agreement with physicians in identifying final diagnoses within the differential-diagnosis lists, achieving a 95.9% agreement rate (236 out of 246 lists; κ=0.86). In contrast, this study demonstrated a fair to good agreement rate of 82.1% (966/1176 lists; κ=0.63). Despite using the same evaluation methods in both studies, the observed decrease in the agreement can be attributed to several factors: the source of case reports (GIM-published vs a broader range of case reports), the generators of differential diagnoses (physicians, GPT-3/GPT-4 vs GPT-4/Google Bard [currently Gemini]/LLaMA2 chatbot), and the volume of lists assessed (246 lists vs 1176 lists).

### Future Directions

Future studies explore the potential of integrating GPT-4 and similar AI systems into real-world clinical settings. This could involve developing interfaces that allow these AI systems to interact directly with electronic health records, providing real-time diagnostic feedback to physicians. Additionally, research could focus on tailoring these AI systems for specialized medical fields, where their ability to process vast amounts of data could significantly aid in complex case analysis. Another vital area for future research is the ethical implications of AI in medicine [43], particularly in patient data privacy, AI decision transparency, and the impact of AI-assisted diagnostics on physician-patient relationships.

Furthermore, further research should also investigate the optimal use of AI technologies, including the exploration of both chatbot interfaces and application programming interface functionalities. A more detailed examination of application programming interface settings, such as adjustable parameters including temperature and Top P, could be invaluable. This investigation would provide clearer guidelines on when and how to use different AI tools effectively, considering both scientific evidence and effectiveness.

Moreover, our future research will focus on refining the evaluation of AI-generated differential diagnoses by incorporating more sophisticated and validated psychometric methods as the next diagnostic step. We propose to adopt methodologies for assessing the quality of differential diagnoses. This approach will allow us not only to compare AI-generated outputs with those from physicians but also to treat it as a form of Turing test—evaluating whether AI can match or surpass human performance in diagnostic tasks without being distinguishable from them [46].

### Conclusions

GPT-4 demonstrated a fair to good agreement in identifying the final diagnosis from differential-diagnosis lists, comparable to physicians for case report series. By reliably identifying diagnoses, GPT-4 can provide on-time feedback by comparing final diagnoses with differential-diagnosis lists. Therefore, this study suggests that generative AI systems have the potential to assist physicians in the diagnostic process by providing reliable and efficient feedback, thereby contributing to improved clinical decision-making and medical education. However, it is imperative to recognize that these findings are based on experimental studies. Real-world scenarios could present unique challenges, and further validations in diverse clinical environments are essential before broad implementation can be recommended.

## Appendix

Multimedia Appendix 1 The differential-diagnosis generated by 3 artificial intelligences used in this study and the final diagnosis.

Multimedia Appendix 2 Structured prompt used in this study.

Multimedia Appendix 3 Formed data set used in this study.

## Abbreviations

AI: artificial intelligence
CDSS: clinical decision support system
GIM: general internal medicine
LLaMA2: LLM Meta AI 2
LLM: large language model
