# Supplementary material for: Evaluating ChatGPT-4’s Accuracy in Identifying Final Diagnoses Within Differential Diagnoses Compared With Those of Physicians: Experimental Study for Diagnostic Cases
Source: JMIR Form Res. 2024 Jun 26;8:e59267. doi: 10.2196/59267 (PMC11237772; doi:10.2196/59267)
Supplement: Multimedia Appendix 2 [file formative_v8i1e59267_app2.pdf]

Supplemental Table 2. Structured prompt utilized in the current study.

The format of the prompt
Make the following structured output and DO NOT alter the final diagnosis and the sequence of the Differential Diagnosis List provided:<br>**<br>Final Diagnosis: (copy & paste the final diagnosis)<br>Differential Diagnosis List (1-10): (copy & paste the lists)<br><br>Assessment:<br>- Was the final diagnosis included in the differential-diagnosis list?<br>- Answer: [0 or 1, 0 for NO, 1 for YES]<br><br>- If yes, at what position in the list was the final diagnosis found?<br>- Position: [(Specify between 1-10) or indicate 0 if not included]<br><br>- Reasons for inclusion or exclusion of the final diagnosis in/from the differential diagnosis list:<br>- Explanation: [Provide a brief rationale here]

****
